# An Audit of Patients Who Did Not Attend Appointments in the East Lancashire Memory Assessment Service

**DOI:** 10.1192/bjo.2025.10656

**Published:** 2025-06-20

**Authors:** Kamran Prihar, Claire-Marie Hosein, Oli Sparasci

**Affiliations:** 1 Lancashire and South Cumbria NHS Foundation Trust, Burnley, United Kingdom; 2 The University of Manchester, Manchester, United Kingdom

## Abstract

**Aims:** It is estimated that the rate of non-attendance at outpatient appointments in the NHS is 7.6%. As such, reducing the number of patients who “Did Not Attend” (DNA) is of paramount importance for improving capacity within the current NHS funding envelope.

The East Lancashire Memory Assessment Service (MAS) leads on cognitive assessment for patients from a geographically large catchment area which includes Hyndburn, Rossendale, Blackburn with Darwen, Burnley, Pendle and the Ribble Valley. Patients undergo a multi-disciplinary assessment, which typically includes a triage, initial assessment and diagnostic appointment. Medication monitoring is offered as required.

This audit aimed to establish how many patients DNA their MAS appointments and to understand the reasons for this.

**Methods:** We audited the records of the last 70 patients who had been discharged from the MAS as of 11 November 2024.

The Electronic Patient Record (EPR) was searched to identify key demographic characteristics and to establish whether any appointments were recorded as having an outcome of DNA. For any appointments that were not attended, we established what type of appointment had not been attended and whether any reminders had been sent.

Excel was used for data collection and analysis. Audit approval was granted by LSCFT.

**Results:** A total of 4 instances of patients not attending appointments were recorded in the EPR. These DNA were attributed to three patients. One who DNA an initial assessment, one who DNA a diagnostic appointment and one who DNA both an initial and a diagnostic appointment. In total, 99 appointments were offered to the patients in the audit sample, giving a DNA rate of 4%.

When there was a recorded reason for non-attendance, transport issues and an acute hospital admission were cited. Two patients sadly died whist awaiting already rescheduled initial assessments (these were not classed as DNA). Of the patients audited, there were no DNA for medication monitoring appointments.

Telephone reminders were offered to the majority of patients, 48 hours prior to their appointment, which may have reduced the total number of DNA. These reminders frequently led to appointments being re-arranged to more convenient times, helping to reduce the DNA rate.

**Conclusion:** Comprehensive telephone reminders ensure that the rate of DNA in the East Lancashire MAS is kept to a minimum and allows appointments to be rescheduled if necessary. This audit has demonstrated that non-attendance at MAS appointments happens due to varied factors that are often outside of NHS control.

